# Cigarette smoking and gray matter brain volumes in middle age adults: the CARDIA Brain MRI sub-study

**DOI:** 10.1038/s41398-019-0401-1

**Published:** 2019-02-11

**Authors:** Martine Elbejjani, Reto Auer, David R. Jacobs, Thaddeus Haight, Christos Davatzikos, David C. Goff, R. Nick Bryan, Lenore J. Launer

**Affiliations:** 1 Laboratory of Epidemiology and Population Science, National Institute on Aging, Bethesda, MD USA; 2 Institute of Primary Health Care (BIHAM), University of Bern, Bern, Switzerland; 3 Division of Epidemiology and Community Health, School of Public Health, University of Minnesota, Minneapolis, MN USA; 4 Department of Radiology, University of Pennsylvania Health System, Philadelphia, PA USA; 5 National Heart, Lung, and Blood Institute, Bethesda, MD USA

## Abstract

Cigarette smoking has been associated with dementia and dementia-related brain changes, notably gray matter (GM) volume atrophy. These associations are thought to reflect the co-morbidity of smoking and vascular, respiratory, and substance use/psychological conditions. However, the extent and localization of the smoking-GM relationship and the degree to which vascular, respiratory, and substance use/psychological factors influence this relationship remain unclear. In the Coronary Artery Risk Development in Young Adults CARDIA cohort (*n* = 698; 52% women; 40% black participants; age = 50.3 (SD = 3.5)), we examined the associations of smoking status with total GM volume and GM volume of brain regions linked to neurocognitive and addiction disorders. Linear regression models were used to adjust for vascular, respiratory, and substance use/psychological factors and to examine whether they modify the smoking-GM relationship. Compared to never-smokers, current smokers had smaller total GM volume (−8.86 cm^3^ (95%CI = −13.44, −4.29). Adjustment for substance use/psychological – but not vascular or respiratory – factors substantially attenuated this association (coefficients = −5.54 (95% CI = −10.32, −0.76); −8.33 (95% CI = −12.94, −3.72); −7.69 (95% CI = −6.95, −4.21), respectively). There was an interaction between smoking and alcohol use such that among alcohol non-users, smoking was not related to GM volumes and among alcohol users, those who currently smoked had −12 cm^3^ smaller total GM, specifically in the frontal and temporal lobes, amygdala, cingulate, and insula. Results suggest a large-magnitude association between smoking and smaller GM volume at middle age, accounting for vascular, respiratory, and substance use/psychological factors, and that the association was strongest in alcohol users. Regions suggested to be most vulnerable are those where cognition and addiction processes overlap.

## Introduction

Cigarette smoking has been associated with higher risk of cognitive decline and dementia in older adults^1–4^. Although the mechanisms are not fully understood, these associations are predominantly thought to arise from accumulated smoking-related damage to cardiovascular and respiratory processes in the brain^1,2,5,6^. Parallel to its established impact on vascular and respiratory functions, tobacco dependence is the most prevalent substance-dependence disorder and frequently co-occurs with psychiatric conditions such as mood and substance use disorders^7–9^. For instance, among persons with alcohol abuse disorder, smoking prevalence has been estimated to be as high as 90%^10,11^.

In line with its documented effects on cardiovascular health, some imaging studies have reported that smoking is related to compromised vascular and circulation processes in the brain, including changes in perfusion and white matter (WM) lesions^4,12–14^. One consistent finding from imaging studies is that smokers have smaller total brain volume^4,12,15^, and smaller gray matter (GM) volume in particular, compared to never-smokers^6,16^. Interestingly, several results show that this association may be differentially localized^16,17^, suggesting that more direct links between smoking and specific brain structures could be underlying the associations of smoking to functional outcomes such as addiction and cognitive functioning.

However, findings on which brain structures may preferentially be affected by smoking are heterogeneous. The discrepancy could be explained by methodological limitations. One limitation of published studies is that they were mostly conducted in small (often fewer than 100 subjects)^15,16^ and selective samples (based on participants with several morbidities)^18,19^. Further, studies in community-dwelling individuals have focused mainly on older populations and thus may have had limited ability to disentangle the primary brain changes associated with smoking from those resulting from general accumulated damage such as advanced forms of cardiovascular disease^4,5,20^. Another main limitation is that prior studies included limited adjustment for important contributing factors, such as cardiovascular, respiratory, and substance use/psychological factors^17^. Given their high co-occurrence and complex relationships with smoking and GM, the role of these factors warrants careful investigation^1,2,5,11,21^.

In this study, we used a community-based sample of middle-aged adults to assess the associations of smoking with total brain GM volume and with volumes of candidate brain regions linked to cognitive impairment and addictive behaviors, while assessing the role of vascular, respiratory, and substance use/psychological factors. We first adjusted for these factors and then tested whether they modified the smoking-GM relationship. We hypothesized that GM volumes would be smaller in smokers, particularly in smokers with additional vascular risk factors and addiction-related behaviors.

## Materials and methods

### Study sample

The Coronary Artery Risk Development in Young Adults (CARDIA) study is a population-based longitudinal study of the determinants of cardiovascular disease in young black and white adults^22^. At baseline (1985–1986), the study recruited 5115 participants aged 18–30 years randomly selected by telephone numbers from census tracts in four US cities (Birmingham, Alabama; Chicago, Illinois; Minneapolis, Minnesota; and Oakland, California); the sample was balanced by race (black, white), sex, and education (high school degree or less, higher than high school). Participants were followed for 25 years (with examinations at year 2, 5, 7, 10, 15, 20, and 25 of follow-up); 72% (*n* = 3499) of the surviving baseline cohort participated in the 25th year examination.

At the 25^th^ year examination, a sub-sample took part in the CARDIA brain magnetic resonance imaging (MRI) sub-study. That sub-study enrolled participants from three CARDIA field centers (Birmingham, Minneapolis, and Oakland) and aimed to achieve a balanced sample within sex-race groups. Exclusion criteria were a contra-indication to MRI or a body size too large for the MRI tube bore. In total, 719 subjects participated in the MRI sub-study.

CARDIA is annually approved by the institutional review boards at each site (the Institutional Review Board of the University of Minnesota, the Kaiser Permanente Northern California Institutional Review Board, University of Pennsylvania Institutional Review Board, The University of Alabama Birmingham Institutional Review Board, and the NIH Office of Human Subjects Research Protection for the Intramural Research Program, National Institute on Aging); separate approval was obtained for the brain MRI study. Each participant provided written informed consent at each exam^12^.

### Brain MRI data

Brain MRIs were acquired using 3-T MR scanners (Oakland: Siemens 3 T Tim Trio/VB 15 platform; Minnesota: Siemens 3 T Tim Trio/VB 15 platform; and Alabama: Philips 3 T Achieva/2.6.3.6 platform). In collaboration with the MRI reading center at the University of Pennsylvania (Section of Biomedical Image Analysis, Department of Radiology), each MRI field center performed the MRI scans and transferred the data to the reading center, according to standardized procedures and following standard quality assurance protocols, previously described^12^. Brain tissue volumes were estimated from the sagittal 3D T1 sequence (Plane Sagittal Coil 12channel File name 3D T1 MPRAGE: Tr 1900 Te 2.89 Fov 250 mm, thickness 1 mm slices 176 slices, Base Res 255, Phase res 100%, Matrix 256 × 256 NSA 1 TI 900 ms Pixel BW 170hz. ETL = 1 Flip = 9).

Image processing, quality control checks, and automated brain tissue volume computations were all performed at the MRI reading center following standardized protocols^12,23–26^. First, all imaging data were converted to Neuroimaging Informatics Technology Initiative (NIFTI) format. Initial quality control (QC) was performed through manual inspection of each subject’s T1 scan for motion artifacts and other quality issues. The preprocessing of the T1-weighted scan involved brain extraction (i.e., the removal of the skull, extra-cerebral tissues and cerebellum) using a multi-atlas segmentation method^27^, correction of image inhomogeneities^28^, and segmentation of the brain parenchyma into GM, WM and cerebrospinal fluid^29^. Brain tissue was segmented into anatomical regions by transferring expert defined region of interest (ROI) labels on a standard template image space to the subject T1 space through non-linear registration^23^. GM and WM were classified into regions of interest according to the Jakob atlas^30^ and further into normal and abnormal and tissue. Within each ROI, GM and WM volumetric measurements are calculated. Total brain volume was obtained by summing GM and WM volumes; total intracranial volume was the sum of GM, WM, and cerebral spinal fluid volumes.

Here, we assessed the associations of smoking with total GM volume, and as a comparison with total brain tissue and total WM volume. We examined associations with lobar GM to assess whether smoking had a global compared to specific association with GM. We also assessed the relationship of smoking with GM volumes in specific brain structures. These regions have been linked to dementia and cognitive impairment and to addiction disorders and have been implicated in memory, learning and cognition as well as emotion and reward processes: hippocampus (memory and emotion regulation), parahippocampal gyrus (memory encoding and recall), entorhinal cortex (memory), precuneus (memory and cognition), cuneus (visual processing and inhibitory control), thalamus (perception, sensory, and motor processes), caudate (motor, learning, and reward processes), putamen (movement and learning), nucleus accumbens (reward), cingulate (emotion and behavior regulation), insula (emotional experience and perception), and the amygdala (affective processing and emotional memory)^21,31–35^.

### Smoking data

Smoking data were collected with the same interviewer-based questionnaire administered at each study examination. Participants were asked if they have ever smoked regularly (defined as at least 5 cigarettes per week, almost every week) for at least three months. If they answered no, they were classified as never-smoker at that study wave. If they answered yes, participants were then asked whether they currently smoked regularly (at least five cigarettes per week, almost every week) for at least three months; participants were classified as current smokers if they answered yes, and as former smokers if they reported no current regular cigarette smoking.

Mid-life smoking status was based on self-reported status (never, former, current) at year 25. This self-report was verified against prior data from earlier study exams; for most participants, self-reported status was consistent across study waves, and for 57 (8.7%) subjects who reported being never-smokers at Year 25, we re-coded them as former-smokers because they had reported being smokers or former-smokers at multiple previous study exams.

### Covariates

All analyses were adjusted for age, race (black/white), educational attainment (≤high school diploma, >high school diploma), total intracranial volume, and study center. We then incorporated data on several factors combined into three groups, representing three mechanisms and pathways that are hypothesized to act as confounders or mediators of the smoking-GM volume relationship: vascular, respiratory, and substance use/psychological factors.

Vascular risk factors included body mass index, history of hypertension (defined as current or prior occurrence of: diastolic blood pressure ≥90 and/or systolic blood pressure ≥ 140mmHg and/or taking antihypertensive medication), cerebro/vascular events (yes/no variable indicating self-reported occurrence of any of the following: heart attack, angina, heart failure, rheumatic heart disease, mitral valve prolapse, stroke and peripheral vascular disease), history of diabetes (defined following ADA criteria for levels of fasting, non-fasting or postprandial OGTT results, HbA1c percent, or use of anti-diabetes medication), and hypercholesterolemia (total cholesterol ≥ 240 mg/DL and/or use of cholesterol lowering medication).

Respiratory factors were history of respiratory illness (defined as current or prior self-reported occurrence of asthma, tuberculosis, chronic bronchitis, chronic obstructive pulmonary disease, emphysema, or use of medication for asthma or for breathing problems), and forced expiratory volume (FEV_1_ estimated at 1 sec, in liters, and measured using a spirometer).

Substance use/psychological factors investigated were self-reported lifetime illicit drug use (yes/no for having ever-used any of the following: marijuana, amphetamines, methamphetamines, cocaine, crack, heroin, or pain-killers for non-medical reasons), depressive symptoms (Center for Epidemiologic Studies – Depression; CES-D) scale^36^, and alcohol consumption, which was based on questions regarding the usual number of alcoholic beverages (wine, beer, or hard liquor) consumed per week; assuming that one drink of beer, wine, or liquor contains 16.7, 17.0, and 19.1 milliliters (mL) of ethanol respectively (as per CARDIA protocol), we computed total consumption per week in mL of ethanol and divided it by 17.24 mL of ethanol per average drink to estimate the usual number of drinks per week. Accordingly, alcohol consumption was classified as no alcohol use (no consumption of alcohol beverages), high-risk alcohol use (based on the sex-specific weekly maximum drinking limits published by the National Institute on Alcohol Abuse and Alcoholism (for men > 14, women > 7 standard drinks/week)), and low-risk alcohol use (>0 and ≤7 drinks for women and >0 and ≤14 drinks for men).

FEV_1_ measures were collected at the year 20 exam, the study visit prior to the MRI. For all other covariates, we used their values at the time of the MRI (year 25 exam).

In sensitivity analyses, we also included adjustment for cancer history (current or previous occurrence of cancer and tumor) and physical activity (measured at year 25 using physical activity scores expressed in exercise units, calculated based on the reported frequency and intensity of participation in 13 categories of moderate and vigorous recreational sports, exercise, leisure, and occupational activities over the prior 12 months)^37^. Since results did not change with these adjustments, data are not shown.

### Statistical analyses

Of the 719 subjects with MRI data, 698 had complete data on smoking and potential covariates, and 643 had data on FEV_1_. Total and regional GM volumes were normally distributed. Multivariable linear regression models were used to examine their relationships with mid-life smoking status at the time of the MRI. In all analyses, we adjusted for basic covariates (age, sex, race, educational attainment, intracranial volume, and study centers). We systematically examined the role of other potentially important covariates by using three models, one adjusting for vascular factors, one for respiratory factors, and one for substance use/psychological factors. Then, we tested, using interaction terms, whether these factors modified the associations of smoking and GM volumes and conducted stratified analyses to examine significant interactions. We primarily focused on other vascular risk factors— which often co-occur with smoking and might influence smoking behavior and its outcomes^6,12,38^— and on alcohol use, given its high co-morbidity with smoking and emerging findings regarding their interactions^11,19,39^.

Given the high prevalence of self-reported ever-use of marijuana (Supplementary Table S1), we assessed in sensitivity analyses, whether there was a change in results after adjusting for or testing for effect modification by the lifetime use (yes/no) of marijuana and by the lifetime extent of marijuana use (the number of times participants reported using marijuana up to study year 25 (classified into ≤2, 3 to 10, 11 to 99, 100 to 499, and ≥500 times)); we saw no significant difference in the conclusions.

In additional analyses, we evaluated the relationship of indicators of smoking history (cumulative pack-years, age at start of smoking, and years of smoking cessation) and GM outcomes among ever-smokers.

All analyses tested two-tailed hypotheses with a significance level of 0.05. Analyses were completed using SAS version 9.3 (SAS Institute, Cary, NC). Data and statistical codes are available from the CARDIA Coordinating Center: http://www.cardia.dopm.uab.edu/contact-cardia.

## Results

Compared to never and former smokers, current smokers were younger and had the highest proportion of black participants, the lowest educational attainment, and the highest prevalence of hypertension (Table 1). Other cardiovascular risk factors were not different across smoking groups (Table 1). Current smokers had the highest frequency of respiratory illnesses and the lowest FEV_1_. All substance use/psychological measures were more frequent in smokers (Table 1): compared to never-smokers, current and former-smokers had higher prevalence of high-risk alcohol drinking and illicit drug use; current smokers had more depressive symptoms.

**Table 1:** Characteristics of the CARDIA-MRI sample by smoking status at Year 25

	Mean (SD) or n (%)		
	Never-smoker *n* = 362	Former-smokers *n* = 219	Current smokers *n* = 117
Age at MRI	50.18 (3.61)	50.82 (3.27)	49.63 (3.57)*
Gender (Women)	189 (52.2%)	120 (54.8%)	57 (48.7%)
Race (Black)	143 (39.5%)	68 (31.1%)	68 (58.1%)*
Educational attainment ( ≤ high school)	48 (13.3%)	52 (23.7%)	49 (41.9%)*
Self-reported history of vascular disease	42 (11.6%)	28 (12.8%)	19 (16.2%)
Hypertension	97 (26.8%)	76 (34.7%)	56 (47.9%)*
Diabetes	35 (9.7%)	22 (10.0%)	16 (13.9%)
Body mass index	28.68 (5.59)	28.86 (5.97)	29.28 (5.80)
High cholesterol	72 (19.9%)	49 (22.4%)	25 (21.4%)
History of respiratory illnesses	51 (14.1%)	42 (19.2%)	33 (28.2%)*
Forced expiratory volume (FEV_1_)^a^	3.12 (0.79)	3.15 (0.77)	2.96 (0.73)
Alcohol consumption			
Non-user	159 (43.1%)	92 (41.6%)	38 (33.0%)
Low-risk alcohol use	167 (46.1%)	85 (38.8%)	42 (35.9%)
High-risk alcohol use	39 (10.77%)	42 (19.2%)	37 (31.6%)*
History of illicit drug use	223 (61.60%)	196 (89.5%)	100 (85.5%)*
Depressive symptoms (CES-D)	8.01 (6.80)	8.73 (6.90)	11.41 (8.36)*

^*^p < 0.01; *p*-values obtained using chi-square tests for comparing categorical variables and ANOVA tests for comparing continuous tests across smoking groups

^a^Forces expiratory volume measured at Year 20 and for *n* = 643 subjects, all other covariate measures at Year 25 and for *n* = 698 subjects

### Smoking and GM volume

Brain volumes are presented in Supplementary Table S2. Compared to never-smokers, current smokers had significantly smaller total GM volume (−8.86 cm^3^ (95% CI = −13.44, −4.29); Table 2). There were no differences in total white matter volume across smoking groups; the trend of smaller total brain volume in smokers reflected the association between smoking and smaller GM (Table 2).

**Table 2:** Smoking status and global brain volumes in the CARDIA brain MRI cohort

	Total brain volume			Total white matter volume			Total gray matter volume		
	Beta	95% CI	*p*	Beta	95% CI	*p*	Beta	95% CI	*p*
**Basic model: demographics, intracranial volume**									
Never-smokers	Ref.			Ref.			Ref.		
Former-smokers	−1.72	−6.55, 3.10	0.49	1.80	−2.25, 5.86	0.38	−3.52	−7.12, 0.07	0.05
Current smokers	**−9.23**	**−15.38**, **−3.09**	**0.003**	−0.37	−5.53, 4.79	0.89	**−8.86**	**−13.44**, **−4.29**	**0.0002**
**Vascular factors model: demographics, intracranial volume, vascular risk factors**									
Never-smokers	Ref.			Ref.			Ref.		
Former-smokers	−1.04	−6.21, 3.41	0.57	1.95	−.118, 6.02	0.35	−3.35	−6.95, 0.25	0.07
Current smokers	**−8.36**	**−14.53**, **−2.19**	**0.008**	−0.030	−5.24, 5.18	0.99	**−8.33**	**−12.94**, **−3.72**	**0.0004**
**Respiratory factors model: demographics, intracranial volume, respiratory factors (respiratory illnesses** **+** **FEV**_**1**_**)**									
Never-smokers	Ref.			Ref.			Ref.		
Former-smokers	−1.58	−6.57, 3.41	0.54	1.58	−2.62, 5.77	0.46	−3.15	−6.88, 0.58	0.10
Current smokers	**−9.06**	**−15.69**, **−2.42**	**0.008**	−1.37	−6.95, 4.21	0.63	**−7.69**	**−6.95**, **4.21**	**0.002**
**Substance use/psychological factors model: demographics, intracranial volume, substance use/psychological factors**									
Never-smokers	Ref.			Ref.			Ref.		
Former-smokers	0.24	−4.78, 5.27	0.93	2.04	−2.23, 6.30	0.35	−1.80	−5.52, 1.93	0.34
Current smokers	−5.24	−11.69, 1.21	0.11	0.30	−5.18, 5.77	0.92	**−5.54**	**−10.32**, **−0.76**	**0.02**

Basic model adjusted for age, sex, education, race, study center, and total intracranial volume, *n* = 698

Vascular factors model adjusted for age, sex, education, study center, race, and total intracranial volume, hypertension, diabetes, history of vascular disorders, BMI, and high cholesterol, *n* = 698

Respiratory factors adjusted for age, sex, education, race, study center, and total intracranial volume, respiratory illness, and FEV_1_, *n* = 643 (sub-sample with spirometer data)

Substance use/psychological factors model adjusted for age, sex, education, race, and total intracranial volume, alcohol consumption, depressive symptoms, and illicit drug use, n = 698

Bold values: We were highlighting the statistically significant results

### Adjustment for vascular and respiratory factors

Adjusting for vascular or respiratory factors slightly attenuated the associations of smoking and total GM volume (with a 6 to 13% change in the smoking-GM volume estimated association; Table 2). There was no indication of interaction between smoking and vascular or respiratory factors in the smoking-GM relationship.

### Adjustment for substance use/psychological factors

The smoking-total GM association was substantially attenuated (from −8.86 to −5.54 cm^3^; 37% change in estimate) when the model included substance use/psychological factors (Table 2).

Similar patterns were found in the lobar (Supplementary Table S3) and candidate region analyses (Supplementary Table S4): Current smokers had significantly smaller GM volume in the temporal, occipital, and frontal lobes, and in the amygdala, entorhinal cortex, cingulate, and insula, compared to never-smokers; these associations were largely attenuated when substance use/psychological factors were taken into account. Former-smokers had a trend for smaller GM volume but they were not significantly different than never-smokers.

Other candidate regions (hippocampus, parahippocampal gyrus, cuneus, precuneus, thalamus, caudate, putamen, and the nucleus accumbens) were not related to smoking status (Supplementary Table S4).

### Interactions between substance use/psychological factors, smoking, and brain volumes

In the substance use/psychological factors model, all factors showed associations with GM volume: high-risk alcohol drinking (coefficient = −5.58 cm^3^ (95% CI = −10.57, −1.14)) and higher CES-D scores (−0.24 cm^3^ (95% CI = −0.46, −0.02) per unit increase in CES-D score) were associated with significantly smaller total GM; illicit drug use was associated with smaller GM (coefficient = −3.69; *p* = 0.07).

Illicit drug use and depressive symptoms did not modify the association between smoking and GM volumes (*p* > 0.3 for interaction terms). There was a significant interaction between alcohol use and smoking indicating that the association is differentially observed in alcohol users (p for interaction of current smoking with high-risk alcohol use = 0.03 and *p* = 0.07 for interaction with low-risk alcohol use). Details regarding the distribution of sample’s characteristics across alcohol use sub-groups are presented in Supplementary Table S5.

### Alcohol use, smoking, and brain volumes

Results of GM volumes across smoking and alcohol use groups (Table 3) show that subjects who don’t use alcohol (irrespective of smoking status) and never-smokers (whether alcohol users or not) do not have different GM volumes (with adjusted mean GM volumes ranging from 516 to 520 cm^3^). However, the combination of current alcohol (both low- and high-risk use) and smoking use was negatively associated with GM volume (Table 3); therefore, we grouped participants into alcohol non-users *vs*. subjects reporting any alcohol use (low-risk and high-risk). Among participants who do not use alcohol, smoking was *not* associated with total or regional GM volumes (Table 4). In contrast, among alcohol users (light and heavy-risk combined), those who currently smoked had −12 cm^3^ smaller total GM volume, and specifically smaller frontal, temporal, amygdala, cingulate, and insula GM volumes, even after accounting for other substance use /psychological, vascular, and respiratory factors (Table 4). There was a trend toward an association of smoking to smaller occipital GM volume (*p* = 0.11). Similar to analyses in the total sample, volumes of other candidate structures of interest were not related to smoking in the stratified analyses (*p* > 0.17; data not shown).

**Table 3:** Brain gray matter volume across alcohol and smoking groups in the CARDIA brain MRI cohort

	Alcohol non-users			Light-risk alcohol users			High-risk alcohol users		
	*n*	Beta^a^ (95% CI)	Mean^b^ (SE)	*n*	Beta^a^ (95% CI)	Mean^b^ (SE)	*n*	Beta^a^ (95% CI)	Mean^b^ (SE)
Never-smokers	156	Ref.	518.44 (1.85)	167	Ref.	520.26 (1.72)	39	Ref.	516.02 (3.54)
Former-smokers	92	−2.32 (−8.79, 4.15)	516.36 (2.38)	85	−0.38 (−6.39, 5.62)	519.69 (2.38)	42	−6.52 (−15.92, 2.88)	512.04 (3.33)
Current smokers	38	3.28 (−5.53, 12.09)	520.50 (3.83)	42	−11.65 (−23.05, −0.26)	511.85 (3.49)	37	−11.11 (−19.60, −2.63)	506.96 (3.91)

^a^Coefficients and 95% confidence intervals (95% CI)

^b^adjusted means and standard errors (SE) estimated using fully-adjusted linear regression models including age, sex, education, race, study center, total intracranial volume, hypertension, diabetes, history of vascular disorders, BMI, high cholesterol, respiratory illness, FEV_1_, depressive symptoms, and illicit drug use

^b^Adjusted means and standard errors

Bold values: We were highlighting the statistically significant results

**Table 4:** Smoking status and total and regional gray matter volumes, stratified by alcohol use, in the CARDIA brain MRI cohort

	Alcohol non-users			Low/high-risk alcohol users		
	β^a^	95% CI	*p*	β^a^	95% CI	*p*
Total GM						
Never-smokers	Ref.			Ref.		
Former-smokers	−2.32	−8.79, 4.15	0.48	−2.69	−7.63, 2.24	0.28
Current smokers	3.28	−5.53,12.09	0.46	**−12.01**	**−18.54**, **−5.48**	**0.0003**
Frontal GM						
Never-smokers	Ref.			Ref.		
Former-smokers	−0.48	−3.31, 2.17	0.72	−1.56	−3.62, 0.49	0.14
Current smokers	1.60	−2.01, 5.21	0.38	**−3.57**	**−6.28**, **−0.85**	**0.01**
Temporal GM						
Never-smokers	Ref.			Ref.		
Former-smokers	−0.48	−2.76, 1.79	0.68	−1.51	−3.25, 0.24	0.09
Current smokers	0.24	−2.86, 3.34	0.88	**−4.14**	**−6.45**, **−1.84**	**0.0005**
Occipital GM						
Never-smokers	Ref.			Ref.		
Former-smokers	−0.57	−2.07, 0.93	0.46	0.66	−0.46, 1.78	0.25
Current smokers	−0.01	−2.06, 2.03	0.99	−1.22	−2.70, 0.27	0.11
Parietal GM						
Never-smokers	Ref.			Ref.		
Former-smokers	−0.04	−1.69, 1.61	0.96	−0.19	−1.49, 1.11	0.77
Current smokers	1.15	−1.10, 3.40	0.32	−0.84	−2.56, 0.88	0.34
Amygdala						
Never-smokers	Ref.			Ref.		
Former-smokers	−0.02	−0.11, 0.07	0.64	−0.02	−0.09, 0.04	0.50
Current smokers	−0.02	−0.14, 0.10	0.75	**−0.10**	**−0.18, −0.01**	**0.03**
Insula						
Never-smokers	Ref.			Ref.		
Former-smokers	−0.13	−0.554 0.30	0.56	−0.16	−0.47, 0.16	0.32
Current smokers	0.34	−0.25, 0.92	0.26	**−0.70**	**−1.12, −0.28**	**0.001**
Entorhinal cortex						
Never-smokers	Ref.			Ref.		
Former-smokers	0.04	−0.06, 0.15	0.43	−0.06	−0.14, 0.02	0.15
Current smokers	−0.10	−0.25, 0.05	0.18	−0.06	−0.17, 0.04	0.26
Cingulate						
Never-smokers	Ref.			Ref.		
Former-smokers	−0.24	−0.89, 0.42	0.48	0.004	−0.52, 0.53	0.99
Current smokers	−0.15	−1.05, 0.75	0.74	**−1.17**	**−1.87, −0.48**	**0.001**

^a^Coefficients estimated using fully-adjusted linear regression models including age, sex, education, race, study center, total intracranial volume, hypertension, diabetes, history of vascular disorders, BMI, high cholesterol, respiratory illness, FEV_1_, depressive symptoms, and illicit drug use

In additional analyses (Supplementary Table S6) investigating the associations of GM volumes to smoking characteristics (cumulative pack-years, age at start of smoking, and years since smoking cessation), the interaction between alcohol use and smoking characteristics were weaker than for smoking status (*p*-values for interaction terms > 0.09); however, in general, associations between smoking characteristics and GM outcomes were more pronounced in alcohol users. Higher cumulative pack-years and younger age at smoking initiation were associated with smaller total GM (*p* = 0.11 and *p* = 0.07, respectively); associations with age at smoking initiation were more pronounced for amygdala and temporal GM volumes (*p* = 0.06 and 0.02, respectively).

## Discussion

In community-dwelling middle-aged adults, current smoking was associated with smaller total and regional GM volumes. Importantly, results suggest that substance use/psychological —but not vascular or respiratory— factors substantially influenced these associations. In particular, the association of smoking and GM volumes was different in alcohol users and non-users: subjects who did not use alcohol showed no association between smoking and GM volumes, whereas among alcohol users, those who smoked had significantly smaller total and regional GM volumes compared to never-smokers. Regionally, this association was observed in the temporal and frontal lobes and in the amygdala, cingulate, and insula.

In line with other reports mainly in smaller samples^40^, we found that current smoking was associated with smaller total GM volume but not WM volume^6,15,16^. This association was specifically observed in alcohol users and the difference in GM volume of −12 cm^3^ in current smokers compared to never-smokers in this sub-group was large: roughly equivalent to a decade difference in age in our sample (age was associated with 1.2 cm^3^ smaller volume per year). Results indicate the importance of co-occurring smoking and alcohol use with respect to GM volumes and identify low-risk and high-risk alcohol users in a community-based sample as an “at-risk” subgroup for the potential negative neurocognitive sequelae of smoking.

It has been hypothesized and shown in older cohorts that vascular and respiratory factors may play an important role in the relationship between cigarette smoking and GM^5^. But in this younger cohort, these factors were not strongly related to concurrent smoking status and did not significantly modify the smoking-GM association. Adjusting for these factors only slightly attenuated the estimates of the smoking-GM association, though greater attenuation was observed in the model adjusted for respiratory factors, notably FEV_1_; this might be expected, as FEV_1_ is likely to be a mediating factor of the actions of smoking^5^. Our results suggest that, at middle-age, substance use/psychological factors are more important for how smoking relates to GM volume. Adding each of depressive symptoms, alcohol use, or illicit drug use alone slightly attenuated the smoking-GM relationship (to −7 or −8 cm^3^), but together these correlated factors (as seen in Table 1 and Table S5) had a substantial influence on the relationship (Table 2). Importantly, the association of smoking with GM varied depending on alcohol use, accounting for depressive symptoms and illicit drug use (Tables 3 and 4). As was found for smoking, there was no evidence for an interaction between alcohol use and illicit drug use or depressive symptoms with regards to GM volume (data not shown) suggesting that the alcohol-smoking interplay might be distinctively noticeable and important for GM volume.

Findings from studies on alcohol use disorder have shown an intricate interplay between smoking and alcohol use. Smoking has been linked to the severity of alcohol abuse and to a lower success rate of recovery interventions^11,41^. Alcohol use disorder has been independently associated with several negative functional and structural brain outcomes, including higher risk of dementia, poorer cognitive function, metabolic abnormalities, and smaller global and regional GM volumes^11,19,41^. Smoking has been found to aggravate some of these outcomes and to suppress metabolic, neuronal, and functional recovery of abstinence interventions^11,41^. Our results complement these findings in adults with alcohol abuse disorder, and show that both low- and high-risk alcohol users in a community-based sample presented adverse associations between smoking and GM volumes.

Regionally, associations were observed with smaller frontal and temporal GM volume in our sample, a finding consistent with other studies of smoking in both younger and older samples^5,16,17,42^, and with studies of other substance use disorders^11,21,43–45^. Smoking was also associated with significantly smaller amygdala, cingulate, and insula volumes. Although the exact mechanisms are not clear, functional and structural changes in the insula, cingulate, and amygdala have been widely documented in cigarette smoking and other addictions^16,17,35,46,47^.We found no associations of smoking with brain structures that have been widely linked to dementia (hippocampus, parahippocampal gyrus, entorhinal cortex, cuneus, and precuneus). There was an association between smoking and smaller entorhinal cortex; but it was not significant after adjustment for substance use/psychological or respiratory factors. Overall, our regional analyses suggest that, at mid-life, smoking seems to be related to emotion and reward/ addiction-related candidate regions and regions at the intersection of cognitive and reward pathways, such as insula, the cingulate, and the frontal and temporal lobes. The frontal, cingulate, and insular regions contain some of the highest densities of nicotinic acetylcholine receptors^48^. Data from animal models suggest that nicotine can have neurotoxic consequences such as cell loss and synaptic alterations^49–51^. Cigarette smoking also involves exposure to several toxic compounds, including carbon monoxide, free radicals, and free oxygen species, which can lead to cellular and oxidative damage directly affecting neuronal and brain tissue^11^ and to compromised inflammatory, respiratory, and vascular systems resulting in deficits in blood oxygen and nutrients to the brain^11,52^. Alcohol use has also been linked to oxidative damage and molecular and cellular impairments^11^; combined smoking and alcohol use could thus make the brain more vulnerable to each substances’ adverse consequences. It is interesting that we found an interaction with alcohol use and not with other substance use. This might be explained by a larger overlap in the involvement of alcohol and smoking with dopaminergic and γ-aminobutryic acid (GABA) and glutamate systems in several brain regions including the thalamus, cingulate, insula, amygdala, and frontal and temporal lobes^53,54^. Additionally, unlike illicit drug use, alcohol use is more frequent and legally and socially accepted, which could lead to different profiles of addiction and users. Further, there could be more potential stigma related to illicit drug use that can influence the accuracy of self-reported use; the information on the extent of use was also limited in our study and might not have well captured this behavior. Finally, with the wider acceptability and availability of alcohol, the interaction observed with alcohol use could also be indicative of more contexts and scenarios facilitating a higher cigarette smoking and exposure.

Combined, results of the risk factors models and the regional GM analyses suggest that behavioral and substance-use mechanisms may be central to smoking behavior at younger age and its relationships to GM outcomes at mid-life. These data are in line with results from recent epidemiological and clinical studies indicating that dual concurrent treatment for smoking and alcohol/substance abuse is more effective for long-term abstinence outcomes than treating each addiction separately^11,41,55^. Importantly, our results suggest that neurobiological links between co-occurring smoking and substance use behaviors with reward-network regions may be underlying the observed synergy between these behaviors in determining the severity of addictive symptoms and relapse.

Given the high prevalence of smoking and alcohol use, their frequent co-occurrence, and the fact that they are modifiable behaviors, our results emphasize the research, clinical, and public health value of further understanding the interplay between these two factors and how it affects brain structures and functions across the life-course and brain aging in late-life.

One limitation of our study is that we cannot exclude the possibility that some brain differences observed between smokers and never-smokers might have preceded and even predisposed to nicotine addiction, due to factors such as early developmental processes related to maternal smoking, a family history of addiction, or exposure to stressors. Our study focused on cigarette smoking and use of other forms of nicotine use was limited (prevalence of cigar, pipe, smokeless tobacco use in our sample was ≤4.3%) or not measured (e.g., e-cigarette and water pipe use) in our sample. The small number of subjects with certain vascular risk factors might have limited the detection of interaction with smoking. Similarly, the low prevalence of the use of specific illicit drugs (other than marijuana use) and the lack of information on actual quantity and duration of use limited our conclusions regarding the role of illicit drug use and of different types of illicit drug use in the relationship of smoking with brain structures. We examined several regions and there is the possibility of false positives. Applying a stringent Bonferroni correction for the 16 regional volumes investigated in the two alcohol subgroups (significance level = 0.0016), the associations with temporal, cingulate, and insula GM remain statistically significant. Overall, the relationships observed were strong and consistent, which supports the overall message that these findings merit further discussion and certainly replication.

In conclusion, in community-dwelling middle-aged adults, current smoking was associated with smaller total and regional GM volumes. These associations were specifically observed in alcohol users, and were localized in the temporal, frontal, insula, and amygdala volumes. Findings suggest important connections between smoking and behavioral co-morbidities and brain regions linked to addiction processes at mid-life. Future research on smoking and brain health, as well as interventions to address smoking and alcohol use, should prioritize subjects who present with both cigarette smoking and alcohol use, as they appear to be at even greater risk for adverse brain outcomes.

## Supplementary information

Supplementary Material
