# Supplementary material for: Cigarette smoking and gray matter brain volumes in middle age adults: the CARDIA Brain MRI sub-study
Source: Transl Psychiatry. 2019 Feb 11;9:78. doi: 10.1038/s41398-019-0401-1 (PMC6370765; doi:10.1038/s41398-019-0401-1)
Supplement: Supplementary file 1 — Supplementary Material [file 41398_2019_401_MOESM1_ESM.docx]

**SUPPLEMENTARY INFORMATION**

**Supplementary Table S1.** Frequency distribution of self-reported non-medical drug use (marijuana and other illicit drug use) by smoking status in the CARDIA brain MRI cohort.

**Supplementary Table S2.** Description (mean and standard deviation) of total brain, white, and gray matter tissue volume as well as lobar gray matter volume and gray matter volumes in candidate regions of cognitive and addiction disorders in the CARDIA brain MRI cohort.

**Supplementary Table S3 and Table S4.** Association of smoking status with gray matter volumes in brain lobes (Table S3) and in candidate brain structures (S4) estimated in four multivariable linear regression models, systematically adjusting for basic demographic factors, vascular risk factors, respiratory factors, and substance/psychological factors.

**Supplementary Table S5.** Characteristics of the CARDA brain MRI cohort by alcohol use status.

**Supplementary Table S6.** Association of smoking characteristics (cumulative pack-years and age at smoking initiation in ever-smokers and years since smoking cessation in former-smokers) with total and regional gray matter volumes in alcohol users and non-users in the CARDIA brain MRI cohort.

**Supplementary Table S1. Illicit drug use and smoking status in the CARDIA brain MRI cohort**

|  | **Never-smokers**  **n=362** | **Former-smokers**  **n=219** | **Current smokers**  **n=117** |
| --- | --- | --- | --- |
| History of total illicit drug use  (marijuana + other illicit drug use) | 223 (61.60%) | 198 (89.60%) | 98 (85.20%) ^a^ |
| History of marijuana use only | 118 (32.60%) | 61 (27.60%) | 26 (22.61%) ^a^ |
| History of other illicit drug use only^c^ | 3 (0.83%) | 5 (2.26%) | 1 (0.87%) ^b^ |

**^a^p<0.0001 for chi-square tests; ^b^p=0.096**

^c^Other illicit drug use defined as use of amphetamines, methamphetamines, cocaine, crack, heroin, or pain-killers for non-medical reasons

**Supplementary Table S2. Brain tissue volumes** **in the CARDIA brain MRI cohort, n=698**

| *Volumes in cm^3^* | **Mean** | **SD** |
| --- | --- | --- |
| **Total brain volume** | 983.88 | 106.35 |
| **Total white matter volume** | 466.17 | 58.92 |
| **Total gray matter volume** | 517.72 | 53.41 |
| **Lobar gray matter volume** | 162.18 | 18.13 |
| Frontal | 132.33 | 14.21 |
| Temporal | 87.02 | 10.29 |
| Parietal | 63.416 | 8.61 |
| Occipital | 983.88 | 106.35 |
| **Candidate regions** |  |  |
| Amygdala | 3.10 | 0.37 |
| Insula | 15.39 | 1.79 |
| Entorhinal Cortex | 2.65 | 0.45 |
| Hippocampus | 6.87 | 0.79 |
| Parahippocampal gyrus | 4.14 | 0.53 |
| Putamen | 9.04 | 1.04 |
| Thalamus | 13.65 | 1.54 |
| Caudate | 7.61 | 0.96 |
| Cuneus | 10.17 | 1.89 |
| Precuneus | 7.08 | 1.46 |
| Cingulate | 27.09 | 3.91 |
| Nucleus Accumbens | 0.90 | 0.15 |

**Supplementary Table S3. Smoking status and lobar GM volume** **in the CARDIA brain MRI cohort**

|  | **Frontal GM** | | | **Parietal GM** | | | **Occipital GM** | | | **Temporal GM** | | |
| --- | --- | --- | --- | --- | --- | --- | --- | --- | --- | --- | --- | --- |
|  | Beta | 95%CI | p | Beta | 95%CI | P | Beta | 95%CI | p | Beta | 95%CI | p |
| **Basic model: demographics, intracranial volume** | | | | | | | | | | | | |
| Never-smokers | Ref. |  |  | Ref. |  |  | Ref. |  |  | Ref. |  |  |
| Former-smokers | -1.64 | -3.12, -0.17 | 0.029 | -0.23 | -1.16, 0.69 | 0.620 | -0.04 | -0.89, 0.82 | 0.932 | -1.08 | -2.33, 0.17 | 0.091 |
| Current smokers | **-2.75** | **-4.63, -0.88** | **0.004** | -0.54 | -1.72, 0.64 | 0.368 | **-1.51** | **-2.59, -0.42** | **0.007** | **-2.53** | **-4.12, -0.93** | **0.002** |
| **Vascular factors model: demographics, intracranial volume, vascular risk factors** | | | | | | | | | | | | |
| Never-smokers | Ref. |  |  | Ref. |  |  | Ref. |  |  | Ref. |  |  |
| Former-smokers | -1.58 | -3.06, -0.10 | 0.037 | -0.18 | -1.11, 0.74 | 0.700 | -0.01 | -0.87, 0.85 | 0.981 | -1.04 | -2.29, 0.22 | 0.105 |
| Current smokers | **-2.60** | **-4.50, -0.70** | **0.007** | -0.45 | -1.63, 0.74 | 0.461 | **-1.41** | **-2.50, -0.31** | **0.012** | **-2.40** | **-4.01, -0.79** | **0.004** |
| **Respiratory factors model: demographics, intracranial volume, respiratory factors (respiratory illnesses + FEV_1_)** | | | | | | | | | | | | |
| Never-smokers | Ref. |  |  | Ref. |  |  | Ref. |  |  | Ref. |  |  |
| Former-smokers | -1.35 | -2.88, 0.18 | 0.084 | -0.39 | -1.35, 0.57 | 0.430 | -0.01 | -0.87, 0.85 | 0.981 | -1.07 | -2.37, 0.24 | 0.108 |
| Current smokers | **-2.24** | **-4.27, -0.20** | **0.031** | 0.15 | -1.42, 1.13 | 0.823 | **-1.18** | **-2.32, -0.03** | **0.044** | **-2.61** | **-4.35, -0.88** | **0.003** |
| **Substance use/psychological** **factors model: demographics, intracranial volume, substance use/psychological** **factors** | | | | | | | | | | | |  |
| Never-smokers | Ref. |  |  | Ref. |  |  | Ref. |  |  | Ref. |  |  |
| Former-smokers | -1.07 | -2.61, 0.46 | 0.170 | 0.02 | -0.95, 0.98 | 0.971 | 0.27 | -0.63, 1.16 | 0.557 | -0.76 | -2.06, 0.54 | 0.252 |
| Current smokers | -1.64 | -3.61, 0.33 | 0.102 | -0.22 | -1.46, 1.02 | 0.730 | -1.00 | -2.15, 0.15 | 0.087 | **-1.76** | **-3.44, -0.09** | **0.039** |

Basic model adjusted for age, sex, education, race, study center, and total intracranial volume, n=698.

Vascular factors model adjusted for age, sex, education, race, study center, and total intracranial volume, hypertension, diabetes, history of vascular disorders, BMI, and high cholesterol, n=698.

Respiratory factors adjusted for age, sex, education, race, study center, and total intracranial volume, respiratory illness, and FEV_1_, n =643 (sub-sample with spirometer data).

Substance use/psychological factors model adjusted for age, sex, education, race, study center, and total intracranial volume, alcohol consumption, depressive symptoms, and illicit drug use, n=698.

**Supplementary Table S4. Smoking status and volume of candidate brain structures in the CARDIA brain MRI cohort**

|  | **Amygdala** | | | **Entorhinal cortex** | | | **Insula** | | | **Cingulate** | | |
| --- | --- | --- | --- | --- | --- | --- | --- | --- | --- | --- | --- | --- |
|  | Beta | 95%CI | p | Beta | 95%CI | p | Beta | 95%CI | p | Beta | 95%CI | p |
| **Basic model: demographics, intracranial volume** | | | | | | | | | |  |  |  |
| Never-smokers | Ref. |  |  | Ref. |  |  | Ref. |  |  | Ref. |  |  |
| Former-smokers | -0.03 | -0.08, 0.02 | 0.182 | -0.03 | -0.08, 0.03 | 0.396 | -0.13 | -0.37, 0.10 | 0.266 | -0.27 | -0.75, 0.22 | 0.275 |
| Current smokers | **-0.08** | **-0.14, -0.02** | **0.010** | **-0.07** | **-0.15, -0.001** | **0.047** | **-0.30** | **-0.60, -0.003** | **0.048** | **-1.10** | **-1.71, -0.48** | **0.0005** |
| **Vascular factors model: demographics, intracranial volume, vascular risk factors** | | | | | | | | | | | | |
| Never-smokers | Ref. |  |  | Ref. |  |  | Ref. |  |  | Ref. |  |  |
| Former-smokers | -0.03 | -0.08, 0.02 | 0.209 | -0.03 | -0.08, 0.03 | 0.394 | -0.14 | -0.38, 0.09 | 0.232 | -0.25 | -0.73, 0.23 | 0.311 |
| Current smokers | **-0.08** | **-0.14, -0.02** | **0.013** | **-0.08** | **-0.15, -0.001** | **0.048** | **-0.31** | **-0.61, -0.016** | **0.039** | **-1.01** | **-1.63, -0.39** | **0.002** |
| **Respiratory factors model: demographics, intracranial volume, respiratory factors (respiratory illnesses + FEV_1_)** | | | | | | | | | | | | |
| Never-smokers | Ref. |  |  | Ref. |  |  | Ref. |  |  | Ref. |  |  |
| Former-smokers | -0.03 | -0.08, 0.02 | 0.239 | -0.02 | -0.08, 0.04 | 0.490 | -0.11 | -0.35, 0.13 | 0.376 | -0.13 | -0.64, 0.38 | 0.614 |
| Current smokers | **-0.08** | **-0.15, -0.01** | **0.018** | -0.07 | -0.15, 0.01 | 0.077 | -0.29 | -0.61 0.03 | 0.075 | **-1.06** | **-1.73, -0.39** | **0.002** |
| **Substance use/psychological factors model: demographics, intracranial volume, substance use/psychological factors** | | | | | | | | | | | | |
| Never-smokers | Ref. |  |  | Ref. |  |  | Ref. |  |  | Ref. |  |  |
| Former-smokers | -0.02 | -0.07, 0.03 | 0.533 | -0.01 | -0.07, 0.05 | 0.706 | -0.11 | -0.35, 0.14 | 0.382 | -0.06 | -0.56, 0.45 | 0.821 |
| Current smokers | -0.04 | -0.11, 0.02 | 0.196 | -0.05 | -0.13, 0.02 | 0.165 | -0.18 | -0.49, 0.13 | 0.262 | **-0.74** | **-1.39, -0.09** | **0.026** |

**Supplemental Table S4 continued. Smoking status and volume of candidate brain structures in the CARDIA brain MRI cohort**

|  | **Hippocampus** | | | **Parahippocampal gyrus** | | | **Cuneus** | | | **Precuneus** | | |
| --- | --- | --- | --- | --- | --- | --- | --- | --- | --- | --- | --- | --- |
|  | Beta | 95%CI | p | Beta | 95%CI | p | Beta | 95%CI | p | Beta | 95%CI | p |
| **Basic model: demographics, intracranial volume** | | | | | | | | | | | | |
| Never-smokers | Ref. |  |  | Ref. |  |  | Ref. |  |  | Ref. |  |  |
| Former-smokers | -0.03 | -0.14, 0.09 | 0.636 | -0.01 | -0.07, 0.06 | 0.806 | -0.13 | -0.36, 0.10 | 0.258 | -0.12 | -0.33, 0.09 | 0.274 |
| Current smokers | -0.11 | -0.25, 0.03 | 0.134 | -0.04 | -0.12, 0.04 | 0.337 | -0.21 | -0.50, 0.09 | 0.165 | -0.19 | -0.47, 0.08 | 0.162 |
| **Vascular factors model: demographics, intracranial volume, vascular risk factors** | | | | | | | | | | | | |
| Never-smokers | Ref. |  |  | Ref. |  |  | Ref. |  |  | Ref. |  |  |
| Former-smokers | -0.03 | -0.14, 0.08 | 0.603 | -0.01 | -0.07, 0.06 | 0.786 | -0.13 | -0.36, 0.10 | 0.255 | -0.13 | -0.35, 0.08 | 0.223 |
| Current smokers | 0.11 | -0.26, 0.03 | 0.135 | -0.04 | -0.12, 0.05 | 0.380 | -0.19 | -0.49, 0.10 | 0.201 | -0.21 | -0.48, 0.06 | 0.132 |
| **Respiratory factors model: demographics, intracranial volume, respiratory factors (respiratory illnesses + FEV_1_)** | | | | | | | | | | | | |
| Never-smokers | Ref. |  |  | Ref. |  |  | Ref. |  |  | Ref. |  |  |
| Former-smokers | 0.01 | -0.11, 0.12 | 0.934 | -0.004 | -0.07, 0.06 | 0.903 | -0.12 | -0.35, 0.12 | 0.335 | -0.05 | -0.27, 0.18 | 0.677 |
| Current smokers | -0.08 | -0.23, 0.08 | 0.350 | -0.01 | -0.10, 0.08 | 0.805 | -0.11 | -0.43, 0.20 | 0.489 | -0.09 | -0.38, 0.21 | 0.561 |
| **Substance use/psychological factors model: demographics, intracranial volume, substance use/psychological factors** | | | | | | | | | | | | |
| Never-smokers | Ref. |  |  | Ref. |  |  | Ref. |  |  | Ref. |  |  |
| Former-smokers | 0.03 | -0.09, 0.15 | 0.603 | 0.002 | -0.07, 0.07 | 0.957 | -0.08 | -0.32, 0.17 | 0.537 | -0.03 | -0.25, 0.20 | 0.809 |
| Current smokers | -0.01 | -0.16, 0.14 | 0.857 | -0.01 | -0.09, 0.08 | 0.863 | -0.15 | -0.46, 0.16 | 0.327 | -0.09 | -0.37, 0.20 | 0.547 |

**Supplemental Table S4 continued. Smoking status and volume of candidate brain structures in the CARDIA brain MRI cohort**

|  | **Thalamus** | | | **Putamen** | | | **Caudate** | | | **Nucleus Accumbens** | | |
| --- | --- | --- | --- | --- | --- | --- | --- | --- | --- | --- | --- | --- |
|  | Beta | 95%CI | p | Beta | 95%CI | p | Beta | 95%CI | p | Beta | 95%CI | p |
| **Basic model: demographics, intracranial volume** | | | | | | | | | | | | |
| Never-smokers | Ref. |  |  | Ref. |  |  | Ref. |  |  | Ref. |  |  |
| Former-smokers | -0.02 | -0.22, 0.19 | 0.882 | -0.08 | -0.21, 0.05 | 0.244 | -0.04 | -0.17, 0.09 | 0.562 | -0.02 | -0.04, 0.01 | 0.118 |
| Current smokers | -0.16 | -0.42, 0.10 | 0.218 | -0.07 | -0.23, 0.10 | 0.443 | 0.08 | -0.09, 0.25 | 0.342 | -0.02 | -0.06, 0.01 | 0.139 |
| **Vascular factors model: demographics, intracranial volume, vascular risk factors** | | | | | | | | | | | | |
| Never-smokers | Ref. |  |  | Ref. |  |  | Ref. |  |  | Ref. |  |  |
| Former-smokers | -0.03 | -0.24, 0.17 | 0.740 | -0.08 | -0.21, 0.05 | 0.213 | -0.04 | -0.17, 0.10 | 0.592 | -0.02 | -0.04, 0.01 | 0.154 |
| Current smokers | -0.18 | -0.43, 0.08 | 0.186 | -0.07 | -0.23, 0.10 | 0.428 | 0.08 | -0.09, 0.25 | 0.333 | -0.02 | -0.05, 0.01 | 0.284 |
| **Respiratory factors model: demographics, intracranial volume, respiratory factors (respiratory illnesses + FEV_1_)** | | | | | | | | | | | | |
| Never-smokers | Ref. |  |  | Ref. |  |  | Ref. |  |  | Ref. |  |  |
| Former-smokers | 0.03 | -0.18, 0.24 | 0.769 | -0.09 | -0.23, 0.04 | 0.173 | -0.04 | -0.17, 0.10 | 0.615 | -0.22 | -0.05, 0.003 | 0.085 |
| Current smokers | -0.17 | -0.45, 0.11 | 0.235 | -0.10 | -0.28, 0.08 | 0.256 | 0.11 | -0.07, 0.29 | 0.230 | -0.26 | -0.06, 0.01 | 0.128 |
| **Substance use/psychological factors model: demographics, intracranial volume, substance use/psychological factors** | | | | | | | | | | | | |
| Never-smokers | Ref. |  |  | Ref. |  |  | Ref. |  |  | Ref. |  |  |
| Former-smokers | 0.001 | -0.21, 0.22 | 0.994 | -0.07 | -0.20, 0.07 | 0.355 | -0.02 | -0.15, 0.12 | 0.829 | -0.01 | -0.04, 0.02 | 0.427 |
| Current smokers | -0.10 | -0.37, 0.18 | 0.478 | -0.02 | -0.20, 0.16 | 0.820 | 0.12 | -0.06, 0.29 | 0.201 | -0.01 | -0.04, 0.02 | 0.616 |

Basic model adjusted for age, sex, education, race, study center, and total intracranial volume, n=698.

Vascular factors model adjusted for age, sex, education, race, study center, and total intracranial volume, hypertension, diabetes, history of vascular disorders, BMI, and high cholesterol, n=698.

Respiratory factors adjusted for age, sex, education, race, study center, and total intracranial volume, respiratory illness, and FEV_1_, n =643 (sub-sample with spirometer data).

Substance use/psychological factors model adjusted for age, sex, education, race, study center, and total intracranial volume, alcohol consumption, depressive symptoms, and illicit drug use, n=698.

.

**Supplementary Table S5. Characteristics of the CARDA brain MRI sample by alcohol use status at year 25**

|  | **Mean (SD) or n (%)** | | | | | |  |  |
| --- | --- | --- | --- | --- | --- | --- | --- | --- |
|  | **No alcohol use**  **n=286** | | **Low-risk use**  **n=294** | | **High-risk use**  **n=118** | | ***p-value^a^*** | ***N*** |
| Age at MRI | 50.24 | (3.41) | 50.11 | (3.65) | 50.85 | (3.42) | 0.15 | 698 |
| Gender (Women ) | 181 | (63.29%) | 124 | (42.18%) | 61 | (51.69%) | <0.0001 | 698 |
| Race (Black) | 141 | (49.03%) | 105 | (35.71%) | 33 | (27.97%) | <0.0001 | 698 |
| Educational attainment (≤high school) | 74 | (25.87%) | 52 | (17.69%) | 23 | (19.49%) | 0.05 | 698 |
| History of vascular disease^b^ | 41 | (14.34%) | 35 | (11.90%) | 13 | (11.02%) | 0.5 | 698 |
| Hypertension | 103 | (36%) | 84 | (28.57%) | 42 | (35.59%) | 0.13 | 698 |
| Diabetes | 40 | (14%) | 22 | (7.48%) | 11 | (9.32%) | 0.03 | 698 |
| Body mass index | 30.032 | (6.471) | 27.853 | (4.966) | 28.386 | (5.166) | <0.0001 | 698 |
| High cholesterol | 61 | (21.33%) | 56 | (19.05%) | 29 | (24.58%) | 0.45 | 698 |
| History of respiratory illnesses^c^ | 57 | (19.93%) | 35 | (11.90%)) | 34 | (28.81%) | 0.0002 | 698 |
| Forced expiratory volume (FEV1) | 2.890 | (0.732) | 3.304 | (0.782) | 3.131 | (0.747) | <0.0001 | 643 |
| Smoking status |  |  |  |  |  |  |  |  |
| Never-smokers | 156 | (54.55%) | 167 | (56.80%) | 39 | (33.05%) | <0.0001 | 698 |
| Former-smokers | 92 | (32.17%) | 85 | (28.91%) | 42 | (35.59%) | 0.39 | 698 |
| Current smokers | 38 | (13.29%) | 42 | (14.29%) | 37 | (31.36%) | <0.0001 | 698 |
| History of illicit drug use | 178 | (62.24%) | 235 | (79.93%) | 106 | (89.83%) | <0.0001 | 698 |
| Depressive symptoms (CES-D) | 9.105 | (7.64) | 8.269 | (6.53) | 9.407 | (7.68) | 0.23 | 698 |

^a^ p-values obtained using chi-square tests for categorical variables and ANOVA tests for continuous tests.

**Supplementary Table S6. Smoking characteristics in ever-smokers and brain gray matter volumes in the CARDIA brain MRI cohort**

|  | **Alcohol non-users**  **ever-smokers, n=130** | | | **Alcohol users**  **ever-smokers, n=206** | | |
| --- | --- | --- | --- | --- | --- | --- |
|  | **ß^a^** | **95% CI** | **p** | **ß^a^** | **95% CI** | **p** |
| **Total GM** |  |  |  |  |  |  |
| Pack years^b^ | -0.10 | -0.43, 0.24 | 0.567 | -0.26 | -0.57, 0.06 | 0.107 |
| Age at start of smoking^c^ | 0.19 | -0.85, 123 | 0.719 | 0.54 | -0.05, 1.12 | 0.071 |
| Years of smoking cessation^d^ | 0.09 | -0.42, 0.60 | 0.724 | -0.13 | -0.47, 0.22 | 0.468 |
| **Frontal GM** |  |  |  |  |  |  |
| Pack years^b^ | 0.004 | -0.13, 0.14 | 0.952 | -0.04 | -0.17, 0.09 | 0.553 |
| Age at start of smoking^c^ | -0.09 | -0.49, 0.32 | 0.670 | 0.08 | -0.16, 0.32 | 0.521 |
| Years of smoking cessation^d^ | 0.08 | -0.14, 0.31 | 0.473 | -0.09 | -0.25, 0.06 | 0.229 |
| **Temporal GM** |  |  |  |  |  |  |
| Pack years^b^ | 0.02 | -0.10, 0.15 | 0.744 | -0.06 | -0.17, 0.05 | 0.264 |
| Age at start of smoking^c^ | 0.20 | -0.19, 0.59 | 0.311 | 0.19 | -0.004, 0.39 | 0.055 |
| Years of smoking cessation^d^ | -0.08 | -0.27, 0.11 | 0.398 | -0.01 | -0.14, 0.12 | 0.907 |
| **Occipital GM** |  |  |  |  |  |  |
| Pack years^b^ | -0.08 | -0.15, 0.002 | 0.056 | -0.04 | -0.11, 0.03 | 0.233 |
| Age at start of smoking^c^ | 0.03 | -0.22, 0.28 | 0.800 | -0.01 | -0.13, 0.12 | 0.923 |
| Years of smoking cessation^d^ | 0.02 | -0.10, 0.15 | 0.735 | -0.02 | -0.10, 0.07 | 0.679 |
| **Parietal GM** |  |  |  |  |  |  |
| Pack years^b^ | 0.01 | -0.08, 0.10 | 0.765 | -0.06 | -0.14, 0.01 | 0.102 |
| Age at start of smoking^c^ | -0.03 | -0.31, 0.26 | 0.862 | 0.11 | -0.04, 0.25 | 0.143 |
| Years of smoking cessation^d^ | 0.02 | -0.12, 0.16 | 0.746 | 0.04 | -0.06, 0.14 | 0.424 |
| **Amygdala** |  |  |  |  |  |  |
| Pack years^b^ | -0.001 | -0.01, 0.004 | 0.761 | -0.002 | -0.01, 0.002 | 0.283 |
| Age at start of smoking^c^ | 0.01 | -0.01, 0.02 | 0.457 | **0.01** | **0.002, 0.02** | **0.015** |
| Years of smoking cessation^d^ | -0.01 | -0.01, 0.002 | 0.130 | -0.001 | -0.01, 0.003 | 0.617 |
| **Entorhinal cortex** |  |  |  |  |  |  |
| Pack years^b^ | -0.003 | -0.01, 0.003 | 0.268 | 0.003 | -0.002, 0.01 | 0.218 |
| Age at start of smoking^c^ | 0.01 | -0.01, 0.25 | 0.522 | 0.002 | -0.01, 0.01 | 0.716 |
| Years of smoking cessation^d^ | -0.005 | -0.01, 0.004 | 0.261 | 0.001 | -0.01, 0.01 | 0.733 |
| **Insula** |  |  |  |  |  |  |
| Pack years^b^ | 0.005 | -0.02, 0.03 | 0.658 | -0.01 | -0.03, 0.01 | 0.549 |
| Age at start of smoking^c^ | -0.01 | -0.08, 0.06 | 0.748 | 0.03 | -0.01, 0.06 | 0.109 |
| Years of smoking cessation^d^ | -0.01 | -0.05, 0.03 | 0.727 | -0.01 | -0.03, 0.02 | 0.613 |
| **Cingulate** |  |  |  |  |  |  |
| Pack years^b^ | -0.03 | -0.07, 0.02 | 0.194 | -0.04 | -0.09, 0.002 | 0.059 |
| Age at start of smoking^c^ | 0.06 | -0.07, 0.20 | 0.360 | 0.07 | -0.01, 0.15 | 0.084 |
| Years of smoking cessation^d^ | 0.05 | -0.02, 0.12 | 0.181 | -0.01 | -0.06, 0.05 | 0.811 |

^a^ Coefficients estimated using fully-adjusted model including age, sex, education, race, study center, and total intracranial volume, hypertension, diabetes, history of vascular disorders, BMI, high cholesterol, depressive symptoms, and illicit drug use, respiratory illness, and FEV_1_.

^b^mean pack-years among ever-smokers = 13.12 (SD=13.90) in alcohol non-users and 10.41 (SD=11.48) in alcohol users.

^c^mean age at smoking initiation among ever-smokers = 17.58 (SD=4.27) in alcohol non-users and 18.30 (SD=5.62) in alcohol users.

^d^mean years since cessation estimated for the former-smokers group: 15.37 (SD=10.96) in alcohol non-users and 18.45 (SD =11.21) in alcohol users.
